# Value of Electronic Health Records Measured Using Financial and Clinical Outcomes: Quantitative Study

**DOI:** 10.2196/52524

**Published:** 2024-01-24

**Authors:** Shikha Modi, Sue S Feldman, Eta S Berner, Benjamin Schooley, Allen Johnston

**Affiliations:** 1 The University of Alabama in Huntsville Huntsville, AL United States; 2 The University of Alabama at Birmingham Birmingham, AL United States; 3 Brigham Young University Provo, UT United States; 4 Department of Information Systems, Statistics, and Management Science The University of Alabama Tuscaloosa, AL United States

**Keywords:** acceptance, admission, adoption, clinical outcome, cost, economic, EHR adoption, EHR, electronic health record, finance, financial outcome, financial, health outcome, health record, hospital, hospitalization, length of stay, margin, moderation analysis, multivariate, operating margin, operating, operation, operational, profit, project management, readmission rate, readmission, total margin, value analysis, value engineering, value management

## Abstract

**Background:**

The Health Information Technology for Economic and Clinical Health Act of 2009 was legislated to reduce health care costs, improve quality, and increase patient safety. Providers and organizations were incentivized to exhibit meaningful use of certified electronic health record (EHR) systems in order to achieve this objective. EHR adoption is an expensive investment, given the resources and capital that are invested. Due to the cost of the investment, a return on the EHR adoption investment is expected.

**Objective:**

This study performed a value analysis of EHRs. The objective of this study was to investigate the relationship between EHR adoption levels and financial and clinical outcomes by combining both financial and clinical outcomes into one conceptual model.

**Methods:**

We examined the multivariate relationships between different levels of EHR adoption and financial and clinical outcomes, along with the time variant control variables, using moderation analysis with a longitudinal fixed effects model. Since it is unknown as to when hospitals begin experiencing improvements in financial outcomes, additional analysis was conducted using a 1- or 2-year lag for profit margin ratios.

**Results:**

A total of 5768 hospital-year observations were analyzed over the course of 4 years. According to the results of the moderation analysis, as the readmission rate increases by 1 unit, the effect of a 1-unit increase in EHR adoption level on the operating margin decreases by 5.38%. Hospitals with higher readmission payment adjustment factors have lower penalties.

**Conclusions:**

This study fills the gap in the literature by evaluating individual relationships between EHR adoption levels and financial and clinical outcomes, in addition to evaluating the relationship between EHR adoption level and financial outcomes, with clinical outcomes as moderators. This study provided statistically significant evidence (*P*<.05), indicating that there is a relationship between EHR adoption level and operating margins when this relationship is moderated by readmission rates, meaning hospitals that have adopted EHRs could see a reduction in their readmission rates and an increase in operating margins. This finding could further be supported by evaluating more recent data to analyze whether hospitals increasing their level of EHR adoption would decrease readmission rates, resulting in an increase in operating margins. Hospitals would incur lower penalties as a result of improved readmission rates, which would contribute toward improved operating margins.

## Introduction

### Overview

The Health Information Technology for Economic and Clinical Health (HITECH) Act of 2009 was legislated to reduce health care costs, improve quality, and increase patient safety [1]. Providers and organizations were incentivized to exhibit meaningful use of certified electronic health record (EHR) systems in order to achieve this objective [1]. The HITECH Act was based on the “triple aim” of health care, which consisted of reducing costs, improving the experience of care, and improving population health, and the HITECH Act contributed to the importance of EHRs [2]. Physicians and hospitals that adopted and used certified EHRs as described in federally defined “meaningful use” criteria were awarded approximately US $27 billion in incentives [3] for eligible providers.

EHR adoption is an expensive investment, given the resources and capital that are invested [4,5]. Due to the cost of the investment, a return on the EHR adoption investment is expected. Usually, a return on adoption is measured by calculating net profit and dividing the net profit by net investment [6]. Calculating a return on investment for EHR adoption requires considering the size of the organization, the extent of the EHR adoption, and profit or improvement in terms of both the financial and clinical outcomes perspectives. Given the complex process of calculating return on investment for EHR adoption, this study evaluates return on investment in terms of how it yields value to the adopting entity. Value from the health care perspective has been described in terms of dollars (financial), productivity (clinical), effectiveness (clinical) [7], cost savings (financial) [8], improvement in clinical decisions (clinical; Rudin et al [9]), supporting triage decisions (clinical; Rudin et al [9]), supporting collaborations among the providers (clinical; Rudin et al [9]), increased productivity (financial and clinical) [9], etc. However, a gap exists in that the return on investment is not analyzed in terms of financial and clinical outcomes combined. Additionally, current literature does not review EHR adoption in terms of level of EHR adoption but rather as a binary variable of “adopted” or “not adopted.” This study addresses these gaps by including a combination of both financial and clinical outcomes in a conceptual model and reviewing EHR adoption in terms of levels of EHR adoption.

The value of health IT, of which EHRs are a subset, can depend on the stakeholder and context [10-12]. Looking at value from the stakeholder perspective, for the hospital, EHR value may be reviewed in terms of improved revenue and reduced cost (outcomes); for patients, value may be to improve health and prevent illness (outcomes); for providers, value may be to reduce errors and provide efficient care (process and outcomes); and for government, it may be to improve population health through timely public health reporting and population well-being (process and outcomes) [7]. Hence, given the frequent use of different outcome categories in the literature used to measure value, this study focuses on outcomes as the main value construct and investigates value in terms of different tangible outcomes, such as financial and clinical outcomes. This study examined how EHR adoption levels are associated with value in terms of financial and clinical outcomes combined in 1 model. To address this question, this study investigated the relationship between EHR adoption levels and financial and clinical outcomes by combining both financial and clinical outcomes into 1 conceptual model.

### Conceptual Framework and Hypotheses

**Figure 1:**
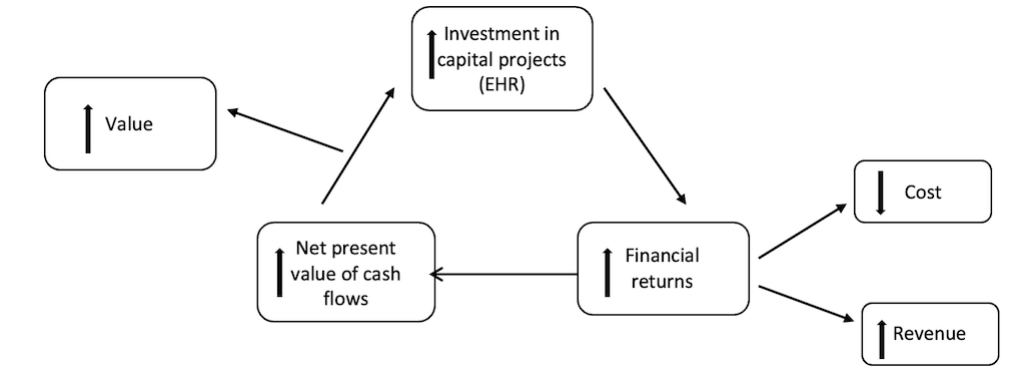
The corporate financial theory of the firm. EHR: electronic health record.

This study used the corporate financial theory of the firm [13] to guide the evaluation of the relationship between EHR adoption and financial and clinical outcomes. The corporate financial theory of the firm (Figure 1) indicates that the value of the firm, or in this case, the health care entity, is expected to be in alignment with the discounted cash flows from the investments, such as EHRs [13]. This theory indicates that a capital investment, such as EHR adoption, increases the value of the firm as it contributes toward an increase in the net present value of cash flows [13]. Multiple studies have supported the notion that EHR investments improve the value of a hospital through improved financial outcomes by way of a reduction in cost or improved revenues [4,14,15].

A study conducted by Collum et al [4] used this theory to determine an association between EHR adoption and financial outcomes (measured as profit margins and return on assets). The findings from this study indicated that financial returns depend on how long it takes for a hospital’s EHR system to achieve full functionality [4], meaning it is important to consider the time variable when reviewing the outcomes of EHR adoption.

Additionally, there have been several studies that have analyzed the relationship between EHR adoption and financial outcomes without using the corporate financial theory of the firm as their guiding framework. Some of the studies from the current literature exhibited a trend that EHR adoption and financial outcomes have a nonlinear relationship [16,17], and some of the studies indicated that EHR adoption resulted in improved financial outcomes for health care organizations that adopted it over time [14,18].

The literature suggests that improvement in costs and revenues is the result of improved clinical outcomes such as reduction of redundant tests [19], reduction of medication and hospital bed-related costs [20], improved ability to capture charges [15], and improved decision support systems [21]. Since this study focuses on combining both financial and clinical outcomes into 1 conceptual model, for the purpose of this study, the capital project investment (EHR adoption in this case) and improvement in financial returns (financial outcomes), tenets of the corporate financial theory of the firm, with an addition of the clinical outcomes, are integrated into a conceptual framework.

**Figure 2:**
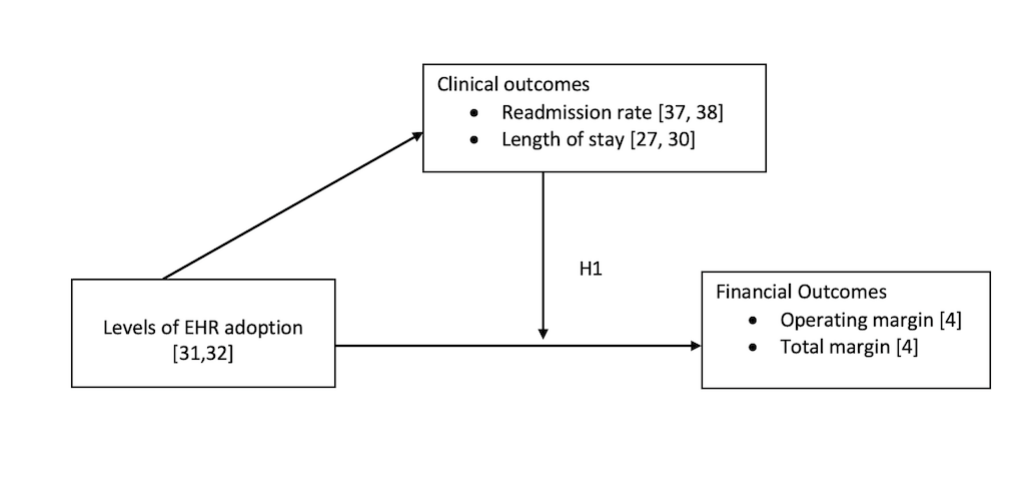
Electronic health record (EHR) value analysis conceptual framework.

The purpose of this conceptual framework (Figure 2 [4,22-27]) is to determine if the previously stated overarching research question of “How is electronic health record adoption associated with value in terms of financial and clinical outcomes?” is supported by the following hypothesis: “The relationship between levels of EHR adoption and financial outcomes (both operating margin and total margin) is moderated by clinical outcomes (readmission rate and length of stay [LOS]) that are also affected by levels of EHR adoption (Figure 2).

## Methods

Data for this study were retrieved from multiple sources, including the Health Care Provider Cost Reporting Information System, the American Hospital Association (AHA) Annual Survey, the AHA IT Supplement Survey, and Health Care Analytics from Leavitt Partners. The study used a longitudinal design from 2014 to 2017 with 5897 hospital-year observations. Measures were divided into 2 groups: financial and clinical. Financial outcome variables were measured or operationalized using 2 variables (operating margin and total margin) that have been used in the health care literature to measure the profitability of hospitals after EHR adoption. The variables describing clinical outcomes are LOS and readmission rates, as these variables have an impact on the financial performance of the hospital [28,29]. The variables describing the financial outcomes are operating margin and total margin, as these measures include both costs and revenues described in the corporate financial theory of the firm [4,13]. The dependent variables used in this study (operating margins, total margins, LOS, and readmission rates) are not comprehensive in terms of measuring financial and clinical outcomes for a hospital; however, for the purpose of this study, these variables are considered sufficient, given their potential association with one another.

### Dependent Variables

#### Financial Outcome Variables

In order to gain an understanding of the financial performance of acute care hospitals, profitability ratios are the most frequently used measures [30]; hence, this study included operating margin and total margin as variables representing financial outcomes. Operating margin captures the expenses and revenues related to hospital operations. Total margin measures or captures operating and nonoperating expenses and revenues. The operating margin was calculated by dividing net patient revenues less total operating expenses by net patient revenues and multiplying the ratio by 100. The total margin variable is calculated by dividing net income by total patient revenue. The financial outcome variables are retrieved from the AHA Annual Survey (2014-2017).

#### Clinical Outcome Variables

Clinical outcomes were measured using LOS and readmission rates. Daniel et al [22] and Schreiber and Shaha [31] reported an intersection of financial and clinical outcomes as a result of EHR adoption and focused on LOS. These studies reported an improvement in LOS due to EHR adoption, resulting in lower plan premiums for patients [22] and costs [31]. Readmission rates are a part of the value-based purchasing program, and depending on the readmission rate, hospitals are penalized on a yearly basis, hence impacting hospital costs [28]. The readmission rates were measured for 6 conditions or procedures, as patients with these conditions are more likely to be readmitted to the hospital. These conditions are: acute myocardial infarction, chronic obstructive pulmonary disease, heart failure, pneumonia, coronary artery bypass graft surgery, and elective primary total hip arthroplasty and total knee arthroplasty [32]. LOS captures the number of days a patient spent in the hospital. Readmission rates indicate whether patients are readmitted to the hospital within 30 days of being discharged. The average LOS and readmission rates can be considered to be indicators of clinical quality outcomes by way of clinical quality measures [28]. Ben-Assuli et al [33] and Lee et al [34] have indicated improvements in average LOS and readmission rates as results of EHR adoption. To confirm these findings for the most recent data, this study analyzes how EHR adoption influences both average (LOS) and readmission rates for the selected sample.

The LOS variable is measured as the average number of days a patient stays in one hospital. The readmission rate variable is measured as the readmission rate payment adjustment factor. The full-year payment adjustment factor is based on data from the fiscal year Hospital Readmissions Reduction Program performance period (ie, July 1, 2014, to June 30, 2017). The minimum payment adjustment factor is 0.97 (ie, 3% maximum penalty). The maximum payment adjustment factor is 1 (ie, no penalty). Hospitals with higher payment adjustment factors have lower penalties [32].

#### Independent Variables

The level of EHR adoption is considered the major explanatory variable in this study. Hospitals are required to report the extent of adoption of each of the 28 EHR functions to the AHA IT Supplement Survey. The 28 EHR functions can be characterized into 5 different categories: clinical documentation, results viewing, computerized order entry, decision support, and bar coding. Hospitals indicate if each function is implemented in all units, 1 unit, or is in some stage of planning. A study conducted by Everson et al [23] emphasizes the reliability and validity of measuring hospital adoption of EHR with these 28 items.

In order to look at the extent of EHR adoption, Adler-Milstein et al [24] created a continuous EHR adoption measure for each hospital in each year in which they responded to the AHA IT Supplement Survey. The continuous measure was constructed as follows: for each function that was implemented in all units, a hospital received 2 points, and for each function that was implemented in at least 1 unit, a hospital received 1 point. According to the calculations, the total possible EHR adoption score ranged from 0 to 56. In order to improve interpretability, the measure was scaled by dividing each hospital’s total score by 56, which yielded an EHR score ranging from 0 to 1. This strategy will be replicated in this study and applied to the EHR adoption level [24].

#### Control Variables

Control variables for this study include time-variant variables such as competition and payer mix. Control variables are identified based on elements that may influence the level of EHR adoption or hospital financial and clinical outcomes [4]. Since this study uses panel data, which accounts for changes in financial outcomes within hospitals due to changes in levels of EHR adoption, it is not essential to control for time-invariant hospital characteristics such as size of the hospital, ownership, system affiliation, and teaching status. For the purpose of this study, time-variant components that may change over the years, such as competition and payer mix, are considered control variables [4].

The competition construct was operationalized using the Herfindahl-Hirschman Index (HHI), which measures the concentration of an industry in a designated market. HHI was measured in terms of discharges for the health service area. Payer mix was measured using the proportion of inpatient days that were related to Medicare and Medicaid patients (Medicare percentage = total facility Medicare days/total inpatient days, and Medicaid percentage = total facility Medicaid days/total inpatient days). The AHA Annual Survey was used to collect the HHI and payer mix data.

### Analysis

The unit of analysis for this study is at the hospital level. To demonstrate the appropriateness of the variables, univariate statistics and bivariate analyses were conducted. Bivariate statistics were generated for both independent and dependent variables of interest. Pairwise correlation analysis was conducted at the significance level of *P*<.05 in order to examine pairwise correlation coefficients between the continuous variables.

Multivariate relationships between different levels of EHR adoption and financial and clinical outcomes, along with the time-variant control variables, were examined using moderation analysis with a longitudinal fixed effects model [35]. Since it is unknown as to when hospitals begin experiencing improvements in financial outcomes, additional analysis was conducted using a 1- or 2-year lag for profit margin ratios [4]. Statistical significance was noted at the significance levels of *P*<.10, *P*<.05, and *P*<.01, and all statistical analyses were conducted in Stata (version 16; StataCorp).

#### Longitudinal Fixed Effects Moderation Analysis Model

A longitudinal fixed effects model with moderation analysis was used to analyze the multivariate relationships between different levels of EHR adoption and financial and clinical outcomes, along with the time variant control variables.

y_it_ = β_1_X_it1_ + β_2_X_it2_ + β_3_ X_it1_ X_it2_ + Z_it_λ + α_i_ + μ_it_

In this equation, y_it_ is the dependent variable (financial or clinical outcomes), i = hospital, and t = time. β_1_ is the coefficient for the main independent variable (levels of EHR adoption), X_it1_. β_2_ is the coefficient for the moderator variable (clinical outcomes), X_it2_. β_3_ is the coefficient for the interaction of the independent variable (levels of EHR adoption) and moderator variable (clinical outcomes), X_it1_X_it2_. Z_it_λ represents all control variables (competition, payer mix, and years of observation). α_i_ is the unknown intercept for a vector of hospitals. And μ_it_ is the error term.

The hypothesis, that the relationship between EHR adoption and financial outcomes is moderated by clinical outcomes, was tested using multiple models. The models and their use are outlined in Textbox 1.

**Analytic models and their use.**

**Model 1**

Determine the association between levels of electronic health record (EHR) adoption and operating margin moderated by length of stay (LOS) with the operating margins from the same year.

**Model 2**

Determine the association between levels of EHR adoption and operating margin moderated by readmission rates with the operating margins from the same year.

**Model 3**

Determine the association between levels of EHR adoption and total margin moderated by LOS with the total margins from the same year.

**Model 4**

Determine the association between levels of EHR adoption and total margin moderated by readmission rates with the total margins from the same year.

**Model 5**

Determine the association between levels of EHR adoption and operating margin moderated by LOS with a 1-year lag in the operating margins.

**Model 6**

Determine the association between levels of EHR adoption and operating margin moderated by LOS with a 2-year lag in the operating margins.

**Model 7**

Determine the association between levels of EHR adoption and operating margin moderated by readmission rates with a 1-year lag in the operating margins.

**Model 8**

Determine the association between levels of EHR adoption and operating margin moderated by readmission rates with a 2-year lag in the operating margins.

**Model 9**

Determine the association between levels of EHR adoption and total margin moderated by LOS with a 1-year lag in the total margins.

**Model 10**

Determine the association between levels of EHR adoption and total margin moderated by LOS with a 2-year lag in the total margins.

**Model 11**

Determine the association between levels of EHR adoption and total margin moderated by readmission rates with a 1-year lag in the total margins.

**Model 12**

Determine the association between levels of EHR adoption and total margin moderated by readmission rates with a 2-year lag in the total margins.

### Ethical Considerations

This study was approved by the University of Alabama at Birmingham institutional review board (300003241).

## Results

Overview

Descriptive statistics of acute care hospitals for the years 2014-2017 are displayed in Table 1. For acute care hospitals, average EHR adoption levels showed little variability across each observed year (approximately 0.89 for each observed year). Hospitals observed a steady decrease in average operating margin from 2014 (0.07%) to 2017 (0.057%). The average total margin across hospitals showed a decrease for 2015 (0.005%) compared with 2014 (1.014%), followed by a steady increase across years 2016 (1.136%) and 2017 (0.951%). An increase in LOS was observed for the years 2016 and 2017 (approximately 7.9 days for the year 2017 vs 3.9 days for the year 2014). Average readmission rates remained somewhat steady across all 4 observation years (approximately 0.99 for each observed year).

**Table 1:** Descriptive statistics of variables (N=5678 hospital-year observations).

Variables			2014 (n=1420)	2015 (n=1453)		2016 (n=1393)		2017 (n=1412)
Levels of EHR^a^ adoption, mean (SD)			0.871 (0.127)	0.890 (0.121)		0.899 (0.127)		0.917 (0.102)
Operating margin, mean (SD)			0.070 (0.122)	0.065 (0.132)		0.06 (0.140)		0.057 (0.136)
Total margin, mean (SD)			1.014 (2.847)	0.005 (26.4)		1.136 (7.217)		0.951 (1.129)
Average length of stay (days), mean (SD)			3.911 (1.134)	3.881 (0.954)		7.87 (153.3)		7.945 (160.4)
Readmission rate, mean (SD)			0.998 (0.003)	0.995 (0.006)		0.995 (0.006)		0.994 (0.007)
Market competition (HHI^b^) in terms of discharges, mean (SD)			0.101 (0.199)	0.086 (0.157)		0.088 (0.172)		0.098 (0.193)
Medicare percentage, mean (SD)			0.512 (0.140)	0.518 (0.128)		0.518 (0.130)		0.521 (0.124)
Medicaid percentage, mean (SD)			0.197 (0.120)	0.202 (0.115)		0.203 (0.114)		0.204 (0.112)
Beds (n), mean (SD)			257 (231)	256 (229)		254 (232)		255 (236)
**Ownership, n (%)**								
	Nongovernment not-for-profit	1105 (77.76 )		1145 (78.8 )	1177 (78.31)		1198 (78.82)	
	Investor-owned for-profit	294 (20.69)		295 (20.30)	311 (20.69)		305 (20.07)	
	Government nonfederal	22 (1.55)		13 (0.89)	15 (1)		17 (1.12)	
**Affiliation, n (%)**								
	Yes	584 (47.29)		660 (51.36)	687 (51.58)		731 (56.67)	
	No	651 (52.71)		625 (48.64)	645 (48.42)		559 (43.33)	
**Teaching status, n (%)**								
	Yes	560 (39.41)		569 (39.16)	595 (39.59)		599 (39.41)	
	No	861 (60.59)		884 (60.84)	908 (60.41)		921 (60.59)	

^a^EHR: electronic health record.

^b^HHI: Herfindahl-Hirschman Index.

For time-variant control variables, the average HHI in terms of discharges across all 4 years was approximately 0.093. HHI values range from 0 to 1, where an HHI value closer to 1 means monopolistic markets, or more market share, and an HHI value closer to 0 means highly competitive markets, or less market share. For the sample used in this study, the markets appear to be highly competitive. In terms of payer mix, the Medicare percentage was similar across all 4 years (average of 0.52). Similarly, the Medicaid percentage was also similar across all 4 years (average of 0.20).

For organizational characteristics, bed size was somewhat similar across all hospitals for all observed years (approximately 255 beds per hospital). In terms of ownership status of the sample hospitals, a majority of the hospitals were nongovernment, not-for-profit hospitals (1105/1421, 78%), followed by investor-owned for-profit hospitals (294/1421, 20%) and government nonfederal hospitals (22/1421, 1.5%). In terms of system affiliation, approximately half the hospitals were affiliated with a system, and the other half were not. For teaching status, a majority of the hospitals did not hold a teaching status (861/1421, 61%).

According to the bivariate statistical analysis (Table 2), at the significance level of *P*<.05, levels of EHR adoption exhibit a positive correlation with operating margin at a magnitude of 0.0978. At the significance level of *P*<.05, readmission rate and levels of EHR adoption are negatively correlated at the magnitude of 0.0321. Even though the magnitudes are close to 0, these relationships are statistically significant at the significance level of *P*<.05.

**Table 2:** Bivariate analysis of variables.

Dependent variables	Independent variables: levels of EHR^a^ adoption (correlation coefficients)
Operating margin	0.0978^b^
Total margin	–0.0142
Average length of stay	0.0039
Readmission rate	–0.0321^b^

^a^EHR: electronic health record.

^b^*P*<.05.

This study tested the following hypothesis that was derived from the EHR value analysis conceptual framework (Figure 2): “The relationship between EHR adoption and financial outcomes is moderated by clinical outcomes.” Tables 3 and 4 provide details relative to the hypothesis.

**Table 3:** Fixed effects with regression analysis.

Variables			Model 1		Model 2		Model 3		Model 4
			OM^a^–LOS^b^–levels of EHR^c^ adoption (Prob>*F*=0.0828)		OM–RR^d^–levels of EHR adoption (Prob>*F*=0.0116)		TM^e^–LOS–levels of EHR adoption (Prob>*F*=0.4532)		TM–RR–levels of EHR adoption (Prob>*F*=0.3388)
Levels of EHR adoption			–0.020		5.335^f^		–4.961		415.2
**Dependent variables**									
	Average LOS	0.000		N/A^g^		–0.002		N/A	
	RR	N/A		4.375^h^		N/A		431.6	
	Levels of EHR adoption and average LOS	-0.000		N/A		0.001		N/A	
	Levels of EHR adoption and RR	N/A		–5.384^f^		N/A		–422.3	
**Control variables**									
	Market competition (HHI^i^)	0.082		0.078		3.148		2.959	
	Medicare percentage	–0.009		–0.013		0.699		0.937	
	Medicaid percentage	–0.026		–0.026		1.211		1.343	
**Years**									
	2014	Reference		Reference		Reference		Reference	
	2015	–0.005		–0.007^f^		–1.001		–0.848	
	2016	–0.008		–0.009^f^		0.388		0.569	
	2017	–0.008		–0.011^j^		0.243		0.460	

^a^OM: operating margin.

^b^LOS: length of stay.

^c^EHR: electronic health record.

^d^RR: readmission rate.

^e^TM: total margin.

^f^*P*<.05.

^g^N/A: not applicable.

^h^*P*<.10.

^i^HHI: Herfindahl-Hirschman Index.

^j^*P*<.001.

**Table 4:** Regression analysis with fixed effects for lagged variables.

Variables			Model 5		Model 6		Model 7		Model 8		Model 9		Model 10		Model 11		Model 12
			OM^a^–LOS^b^–levels of EHR^c^ adoption with 1-year lag (Prob>F=0.0047)		OM–LOS–levels of EHR adoption with 2-year lag (Prob>F=0.0271)		OM–RR^d^–levels of EHR adoption with 1-year lag (Prob>F=0.0010)		OM–RR–levels of EHR adoption with 2-year lag (Prob>F=0.0202)		TM^e^–LOS–levels of EHR adoption with 1-year lag (Prob>F=0.6885)		TM–LOS–levels of EHR adoption with 2-year lag (Prob>F=0.6738)		TM–RR–levels of EHR adoption with 1-year lag (Prob>F=0.6492)		TM–RR– levels of EHR adoption with 2-year lag (Prob>F=0.5143)
Levels of EHR adoption			0.022		0.004		1.681		2.229		1.564		–1.547		–164.0		268.8
Average LOS			0.000		–9.46e–06		N/A^f^		N/A		0.001		–0.012		N/A		N/A
RR			N/A		N/A		0.818		2.192		N/A		N/A		–186.4		169.8
Levels of EHR adoption and average LOS			–0.000		–4.26e–06		N/A		N/A		–0.002		0.013		N/A		N/A
Levels of EHR adoption and RR			N/A		N/A		–1.663		–2.232		N/A		N/A		166.2		–271.7
Market competition (HHI^g^)			0.219^h^		–0.068		0.223^h^		–0.068		3.610		–3.275		3.749		–3.103
Medicare percentage			0.063^i^		0.024		0.068^h^		0.030		–2.419		0.523		–2.416		0.783
Medicaid percentage			0.018		0.891^h^		0.018		0.092^h^		1.742		–2.822		1.741		–2.838
**Years**																	
	2014	Reference		Reference		Reference		Reference		Reference		Reference		Reference		Reference	
	2015	0.014^h^		–0.006		0.014^h^		–0.006		–1.057		–0.714		–1.178		–0.910	
	2016	0.011^h^		–0.008^i^		0.009		–0.008^i^		0.141		0.482		0.048		0.323	
	2017	0.010^h^		–0.018^j^		0.009^i^		–0.018^h^		0.008		0.508		–0.126		0.229	

^a^OM: operating margin.

^b^LOS: length of stay.

^c^EHR: electronic health record.

^d^RR: readmission rate.

^e^TM: total margin.

^f^N/A: not applicable.

^g^HHI: Herfindahl-Hirschman Index.

^h^*P*<.05.

^i^*P*<.10.

^j^*P*<.001.

### EHR: Length of Stay (Operating Margin and Total Margin)

Model 1 analyzed the relationship between EHR adoption levels and operating margins without any lags in operating margins, with LOS as a moderating variable for acute care hospitals. For Model 1, the prob>F was greater than 0.05, meaning this model did not provide a statistical explanation for the proposed relationship between EHR adoption levels and operating margins with LOS as a moderating variable.

Model 5 analyzed the relationship between EHR adoption levels and operating margins with a 1-year lag in operating margins, with LOS as the moderating variable for acute care hospitals. The prob>F was less than .05 for this model; however, the analysis did not provide statistically significant evidence to support the relationship between EHR adoption levels and operating margins with a 1-year lag in operating margins, with LOS as a moderating variable. The nonsignificant results indicated a direct positive association between EHR adoption levels and operating margins and LOS; however, when LOS acts as a moderating variable, the indirect relationship between EHR adoption levels and operating margins was negative.

Model 6 analyzed the relationship between EHR adoption levels and operating margins with a 2-year lag in operating margins, with LOS as a moderating variable for acute care hospitals. Even though the prob>F was less than .05 for this model, the analysis did not provide statistically significant evidence to support the relationship between EHR adoption levels and operating margins with a 1-year lag in operating margins, with LOS as a moderating variable. The nonsignificant results indicated a direct positive association between EHR adoption levels and operating margins with a 2-year lag, which was expected. Additionally, the nonsignificant results indicated a direct negative association between EHR adoption levels and LOS, which is consistent with the findings from the literature. However, when LOS is introduced as a moderating variable, the nonsignificant results indicate a negative indirect relationship between EHR adoption levels and operating margins with a 2-year lag.

Model 3 analyzed the relationship between EHR adoption levels and total margins without any lags in total margins, with LOS as a moderating variable for acute care hospitals. The prob>F was greater than 0.05, meaning the models did not provide a statistically significant explanation for the proposed relationship between EHR adoption levels and total margins without any lags in total margins, with LOS as a moderating variable.

Model 9 analyzed the relationship between EHR adoption levels and total margins with a 1-year lag in total margins, with LOS as a moderating variable for acute care hospitals. For Model 9, the prob>F was greater than 0.05, meaning this model could not accurately predict the relationship between EHR adoption levels and total margins with a 1-year lag in total margins, with LOS as a moderating variable.

Model 10 analyzed the relationship between EHR adoption levels and total margins with a 2-year lag in total margins, with LOS as a moderating variable for acute care hospitals. For Model 10, the prob>F was greater than 0.05, which indicates that this model could not accurately predict the relationship between EHR adoption levels and total margins with a 2-year lag in total margins, with LOS as a moderating variable.

### EHR: Readmission Rate (Operating Margin and Total Margin)

Model 2 analyzed the relationship between EHR adoption levels and operating margins without any lags in operating margins, with readmission rates as a moderating variable for acute care hospitals. Hospitals with higher readmission payment adjustment factors have lower penalties [32]. This was the only model in which the results from the analysis provided statistically significant evidence to support the proposed relationship. At the significance level of *P*<.05, EHR adoption levels were positively associated with operating margins. Similarly, at the significance level of *P*<.05, readmission rates were positively associated with an increase in operating margin. However, when readmission rates are introduced as a moderating variable, the magnitude of the relationship between levels of EHR adoption and operating margins is negative.

Model 7 analyzed the relationship between EHR adoption levels and operating margins with a 1-year lag in operating margins, with readmission rates as a moderating variable for acute care hospitals. For Model 7, the prob>F was less than .05 for this model; however, the analysis did not provide statistically significant evidence to support the relationship between EHR adoption levels and operating margins with a 1-year lag in operating margins, with readmission rates as a moderating variable. The nonsignificant results indicate a direct positive association between EHR adoption levels and operating margins with a 1-year lag and readmission rates, which is consistent with the findings from Model 2. However, when readmission rates act as a moderating variable, the nonsignificant results indicate a positive relationship between levels of EHR adoption and operating margins with a 1-year lag, which was the opposite of the results from Model 2.

Model 8 analyzed the relationship between EHR adoption levels and operating margins with a 2-year lag in operating margins, with readmission rates as a moderating variable for acute care hospitals. The prob>F was less than .05 for this model; however, the analysis did not provide statistically significant evidence to support the relationship between EHR adoption levels and operating margins with a 2-year lag in operating margins, with readmission rates as a moderating variable. Similar to Model 7, the nonsignificant results indicated a direct positive association between EHR adoption levels and operating margins with a 2-year lag and readmission rates, which was consistent with the findings from Model 2. However, when readmission rates act as a moderating variable, the nonsignificant results indicated a positive relationship between levels of EHR adoption and operating margins with a 2-year lag, which was the opposite of the results from Model 2.

Model 4 analyzed the relationship between EHR adoption levels and total margins without any lags in total margins, with readmission rates as a moderating variable for acute care hospitals. The prob>F was greater than 0.05, meaning this model could not provide a statistically significant explanation for the proposed relationship between EHR adoption levels and total margins without any lags in total margins, with readmission rates as a moderating variable.

Model 11 analyzed the relationship between EHR adoption levels and total margins with a 1-year lag in total margins, with readmission rates as a moderating variable for acute care hospitals. For Model 11, the prob>F was greater than 0.05, which indicates that this model could not accurately predict the relationship between EHR adoption levels and total margins with a 1-year lag in total margins, with readmission rates as a moderating variable.

Model 12 analyzed the relationship between EHR adoption levels and total margins with a 2-year lag in total margins, with readmission rates as a moderating variable for acute care hospitals. The prob>F was greater than 0.05, meaning this model could not provide a statistically significant explanation for the proposed relationship between EHR adoption levels and total margins with a 2-year lag in total margins, with readmission rates as a moderating variable.

Results from the regression analysis with fixed effects are displayed in Tables 3 and 4. Table 3 includes results from the regression analysis with financial and clinical outcomes from the same year. Hospitals receive their reimbursement and penalties associated with readmission rates and LOS approximately 1 to 2 years after the actual outcomes occur. In order to accommodate this situation, operating margin and total margin ratios were calculated with a 1- and 2-year lag. Table 4 presents results with lags in profit margins for acute care hospitals.

The results from Table 3 for model 2 suggest that, at the significance level of *P*<.05, a 1-unit increase in EHR adoption was associated with an increase of approximately 5.34% in the operating margin.

Table 4 displays results from the analyses with the added lag effect in operating and total margins. According to the results displayed in Table 4, it can be inferred that at the significance levels of *P*<.05, *P*<.10, or *P*<.001, there is not enough evidence to support models 5-8 from this study. For models 9-12, the models did not provide a statistical explanation for the proposed relationships. In other words, the models discussed above could not accurately predict the proposed relationships.

## Discussion

### Overview

The objective of this study was to determine how EHR adoption level contributes to financial and clinical outcomes for acute care hospitals.

To understand the relationship between EHR adoption level and financial outcomes, moderated by clinical outcomes, this study used a fixed effects moderation analysis model. We hypothesized that there would be a positive association between EHR adoption level and operating and total margins, with LOS and readmission rates as moderating variables.

According to the results displayed in Table 3, for models 1, 3, and 4, the prob>F was greater than .05, meaning the models did not provide a statistical explanation for the proposed relationships in these models. In other words, the models discussed above could not accurately predict the proposed relationships, and there is no evidence that EHR adoption levels have a linear relationship with or explain variance in the operating margins, total margins, and LOS.

Even though the results are inverse of what was predicted in the hypothesis, these findings indicated that the relationship between EHR adoption levels and operating margins was statistically significant when it was moderated by the readmission rates variable at the significance level of *P*<.05. According to the results of the moderation analysis, as the readmission rate increases by 1 unit, the effect of a 1-unit increase in EHR adoption level on the operating margin decreases by 5.38%. In other words, when the hospital incurred lower penalties for readmissions, the operating margins increased. The minimum payment adjustment factor is 0.97 (ie, 3% maximum penalty). The maximum payment adjustment factor is 1 (ie, no penalty), and hospitals with higher payment adjustment factors have lower penalties and, in turn, larger operating margins [32].

In order to confirm any lagged effect (the timeline of hospitals receiving penalties or incentives for EHR adoption being not clear), this study included additional models that accounted for 1- and 2-year lag in the profit margin ratios (models 5-12). The results, however, did not provide any statistically significant evidence supporting a positive relationship between EHR adoption level and profit margin ratios when the lag effect was included in the model.

Findings from current literature indicate an improvement in LOS as a result of EHR adoption (not necessarily adoption level) yielding increased compensation for the loss of patient days from Center for Medicare and Medicaid Services [25]; however, for this study, none of the tested models provided a statistical explanation for the proposed relationships between EHR adoption and profit margins with LOS as moderating variables.

**Figure 3:**
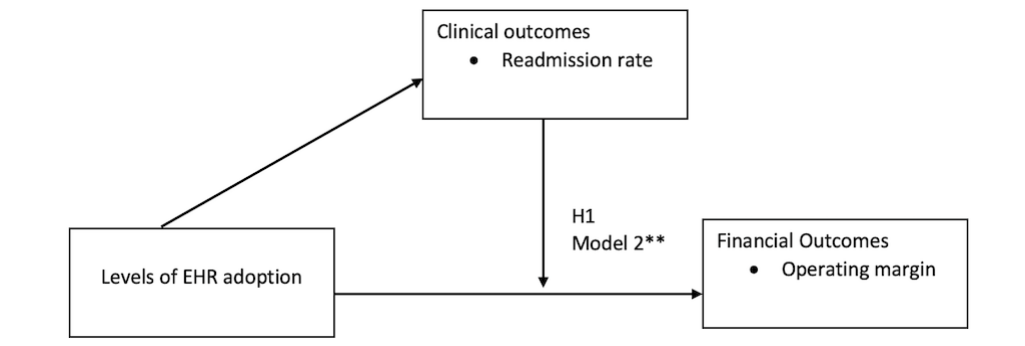
Electronic health record (EHR) value analysis framework with results. **P<.05.

Even though this finding is opposite of what was proposed in the hypothesis, this finding provides statistically significant evidence that levels of EHR adoption change operating margins when readmission rates are taken into account (Figure 3). Analyzing more recent data could indicate a decrease in readmission rates as a result of increased levels of EHR adoption, yielding an increase in operating margins. The relationship between EHR adoption level and operating margins has not been previously evaluated using readmission rates as moderating variables. Hence, this finding from this study is a unique contribution to the current literature.

### Limitations of This Study

Regardless of the valuable contribution of the buildout of the conceptual model and the results from the analysis, this study has limitations. First, there is always a risk when using secondary data to conduct research that was not the intent when the data were collected, as this could result in inconsistency in the data collection methods due to the possibility of human error [36].

Second, this study used data from the Medicare Cost Reports to operationalize the readmission rate variable. This particular measure is reported on a 3-year rolling basis, meaning the data analyzed included a rolling average of 3 years of readmission rate data for each hospital [32]. This study operationalized the readmission rate data for specific years in order to evaluate their relationship with levels of EHR adoption and financial outcomes, which can be considered a limitation.

### Conclusion

The HITECH Act has played an important role in EHRs becoming an integral part of the modern health system over the last 10 years. The goal of enacting the HITECH Act of 2009 was to reduce health care costs, improve the quality of the care provided, and increase patient safety for providers and organizations that exhibited meaningful use of certified EHR systems [1,37]. Given the cost and complexity of EHR adoption, analyzing its value from various and seemingly atypical perspectives is essential.

The current literature does a good job of providing perspectives on EHR value relative to individual financial and clinical outcomes, but it falls short in providing a collective value analysis. This study fills the gap in the literature by evaluating individual relationships between EHR adoption levels and financial and clinical outcomes, in addition to evaluating the relationship between EHR adoption level and financial outcomes, with clinical outcomes as moderators.

This study provided statistically significant evidence, indicating that there is a relationship between EHR adoption level and operating margins when this relationship is moderated by readmission rates. This finding could further be supported by evaluating more recent data to analyze whether hospitals increasing their level of EHR adoption would decrease readmission rates, resulting in an increase in operating margins. Hospitals would incur lower penalties as a result of improved readmission rates, which would contribute toward improved operating margins.

## Abbreviations

AHA: American Hospital Association
EHR: electronic health record
HHI: Herfindahl-Hirschman Index
HITECH: Health Information Technology for Economic and Clinical Health
LOS: length of stay
